# The incidence rate of tuberculosis and its associated factors among HIV-positive persons in Sub-Saharan Africa: a systematic review and meta-analysis

**DOI:** 10.1186/s12879-023-08533-0

**Published:** 2023-09-18

**Authors:** Temesgen Gebeyehu Wondmeneh, Ayal Tsegaye Mekonnen

**Affiliations:** 1 Department of Public Health, College of Health Science, Samara University, Samara, Ethiopia; 2 Department of Biomedical, College of Health Science, Samara University, Samara, Ethiopia

**Keywords:** Incidence, Tuberculosis, HIV, Person, Sub-Saharan Africa

## Abstract

**Background:**

Tuberculosis, along with HIV, is the leading cause of mortality and morbidity globally. Despite the fact that several primary studies have been conducted on the incidence rate of tuberculosis in HIV-infected people in Sub-Saharan Africa, the regional-level tuberculosis incidence rate remains unknown. The objective of this study is to determine the tuberculosis incidence rate and its associated factors in HIV-infected people in Sub-Saharan Africa.

**Methods:**

A systematic review and meta-analysis were conducted by searching four databases for studies published in English between January 1, 2000, and November 25, 2022. The study was carried out using the Preferred Reporting Items for Systematic Reviews and Meta-Analyses (PRISMA) method. To assess the quality of the studies, the Joanna Briggs Institute critical appraisal checklist was used. A random-effects model meta-analysis was used to determine the pooled incidence of tuberculosis using STATA version 15. The I^2^ heterogeneity test was used to assess heterogeneity. Subgroup and sensitivity analyses were performed. Funnel plots and Egger’s regression tests were used to investigate publication bias. The pooled estimate predictors of tuberculosis incidence rate with a 95% confidence interval were also determined using the hazard ratio of each factor (HR).

**Results:**

Out of a total of 3339 studies, 43 were included in the analysis. The overall pooled incidence rate of tuberculosis in HIV-infected people was 3.49 per 100 person-years (95% CI: 2.88–4.17). In the subgroup analysis, the pooled incidence rate of tuberculosis in HIV-infected children was 3.42 per 100 person-years (95% CI: 1.78, 5.57), and it was 3.79 per 100 person-years (95% CI: 2.63, 5.15) in adults. A meta-analysis revealed that underweight (AHR = 1.79, 95% CI: 1.61–1.96), low CD4 count (AHR = 1.23, 95% CI: 1.13–1.35), male gender (AHR = 1.43, 95% CI: 1.22–1.64), advanced WHO clinical stages (AHR = 2.29, 95% CI: 1.34–3.23), anemia (AHR = 1.73, 95% CI: 1.34–2.13), bedridden or ambulatory (AHR = 1.87, 95%), lack of isoniazid preventive therapy (AHR = 3.32, 95% CI: 1.08–2.28), and lack of cotrimoxazole (AHR = 1.68, 95% CI: 1.08–2.28) were risk factors for tuberculosis incidence. HIV patients who received antiretroviral therapy had a 0.53 times higher risk of acquiring tuberculosis than HIV patients who did not receive antiretroviral therapy (AHR = 0.53; 95% CI: 0.3–0.77).

**Conclusion:**

In this systematic review and meta-analysis study, the incidence rate of tuberculosis among HIV-positive people was higher than the WHO 2022 Africa regional estimated report. To reduce the incidence of tuberculosis among HIV patients, HIV patients should take isoniazid prevention therapy (IPT), cotrimoxazole prophylaxis, and antiretroviral therapy (ART) without interruption, as well as increase the frequency and diversity of their nutritional intake. Active tuberculosis screening should be increased among HIV-infected people.

**Supplementary Information:**

The online version contains supplementary material available at 10.1186/s12879-023-08533-0.

## Background

Tuberculosis is caused by Mycobacterium tuberculosis [1, 2]. Tuberculosis remains a major global public health concern [3–5]. Infections with tuberculosis (TB) and human immunodeficiency virus (HIV) interact and influence each other’s pathogenesis [6]. Tuberculosis is the leading cause of death in AIDS patients. Approximately 10.0 million people worldwide developed active tuberculosis (TB), resulting in 1.4 million deaths in 2019 [7]. HIV infection is the strongest known risk factor for Mycobacterium tuberculosis infection and development into active disease, increasing the risk of latent TB reactivation [8]. The HIV infection epidemic has been followed by a serious tuberculosis epidemic [9]. HIV-positive people accounted for 13% of the 8.6 million people with TB in 2012 [10], 12% of the 9.6 million new TB cases in 2014 [11]. In 2018, an estimated 37.9 million people worldwide were infected with HIV, with 1.7 million new infections [12]. Sub-Saharan Africa accounted for 75% of the global HIV/AIDS prevalence [13]. The tuberculosis incidence rate increased by 36% in 2021 compared to 2020, indicating a reversal of the previous two decades’ trend of a nearly 2% decrease per year [14]. In high-burden countries, tuberculosis incidence rises in early adulthood. In low-burden countries, tuberculosis is more common in the elderly and immigrant populations. Young children with tuberculosis are generally less infectious, and data on the tuberculosis disease burden suffered by children has not been systematically collected due to the difficulty of confirming a tuberculosis diagnosis in this age group [15]. Tuberculosis occurred at a rate of 1 case per 100 000 person-years in the US [16], 4.69 cases per 1000 person-years in North America and Europe [17], 0.12 and 0.65 cases per 100 person-year in western and eastern Europe [18], respectively, and 1.54 cases per 100 person-years in Spain [19]. In the United Kingdom (UK), the incidence rate of tuberculosis among people receiving HIV care at UK Collaborative HIV Cohort sites was 1.3 per 1000 person-years [20] and 2.81 cases per 1000 patient-years in tuberculosis-low prevalence settings [21]. The tuberculosis incidence rate was 4.1 per 100 patient-year in Brazil [22] and 750 per 100,000 persons-years in Thailand [23]. In high or intermediate tuberculosis burden settings, the incidence rate of tuberculosis was 4.17 per 100 person-years, whereas in low-burden settings, it was 0.4 per 100 person-years [24]. According to the WHO global tuberculosis (TB) report, Africa had an estimated 212 total tuberculosis incidences and 42 HIV-positive tuberculosis incidence rates per 100,000 populations in 2021 [25]. In Sub-Saharan Africa, the incidence rate among children and adolescents was 2,017 cases per 100,000 patient-years [26], and the incidence rate among HIV-infected patients before and after starting combined antiretroviral therapy was 10.5 per 100 person-years and 5.4 per 100 person-years, respectively [27]. In a large South African multicenter cohort study, the incidence rate of tuberculosis in HIV-positive children receiving antiretroviral therapy was 4.0 cases per 100 person-years [28]. Opportunistic lung infections were more common at CD4 count levels below 200 cells/µl [29–31]. Malnutrition and HIV infection weaken immunity, increasing tuberculosis reactivation and primary progressive disease [32]. The CD4 count is a proxy indicator of disease severity that corresponds to functional status, which is also positively correlated with disease stage [33]. Anemia, defined as hemoglobin levels less than 10 g/dl was found to be strongly and consistently associated with HIV disease progression, as measured by the presence of an AIDS-defining opportunistic disease [34–36]. Advanced WHO clinical stage, bedridden or ambulatory, alcohol consumption [37, 38], lack of antiretroviral therapy [39, 40], underweight (BMI < 18.5 kg/m^2^) [37, 41], male gender, having tuberculosis history [42, 43], and failure to receive isoniazid prevention therapy [44, 45] were risk factors for tuberculosis occurrence. Other factors that contributed to the development of tuberculosis were being married, living in a town [46], having a high number of people in the household [47], having high viral loads [48] and having low ART adherence [38]. Users of cotrimoxazole prophylaxis [37, 49] and nonsmokers [50, 51] were less likely to develop tuberculosis. Despite the fact that global tuberculosis control efforts were off track prior to the COVID-19 pandemic, the COVID-19 pandemic, surprisingly, displaced TB as the leading infectious disease cause of mortality worldwide [7].

As this ongoing disease burden has serious implications in low-income countries, the study’s objective is to determine new tuberculosis occurrences, which indicate current disease transmission, as well as contributing factors that can be used to develop tuberculosis (TB) control programs at the community and regional levels.

## Methods

### Reporting and protocol registration

This systematic review and meta-analysis were reported in accordance with the PRISMA guidelines for reporting systematic reviews and meta-analyses (PRISMA) [52] (S1 Table). The International Prospective Register of Systematic Reviews (PROSPERO) registered this study’s protocol, which is available with the registration number CRD42020176406.

### Search strategies

We used search engines (PubMed, CINHAL, Google Scholar, African journals online, and free Google search databases) in parallel, using search strings adapted to the requirements of each database. We conducted a PubMed search using the following MeSH terms: (i) incidence; (ii) tuberculosis; (iii) HIV infections; (iv) persons; and (v) Africa south of the Sahara, with all qualifiers. The PubMed search was restricted to papers describing human studies published in English between January 1, 2000, and November 25, 2022, because identifying new infections and associated etiologic factors prior to 2000 has not been beneficial for the current prevention programmes. We use the following subject terms for CINHAL databases: (i) incidence; (ii) tuberculosis; (iii) HIV infections; and (iv) persons limited to Sub-Saharan Africa, the English language, and publication dates ranging from January 1, 2000, to November 25, 2022, with open access full text. For Google Scholar, the following keywords were searched: incidence (“incidence rate”), tuberculosis, “HIV-infected persons,“ and “Sub-Saharan Africa,“ limited by the English language and publication year from 2000 to 2022. For African journals online, in the Google custom search text box, we enter the following specific search terms: incidence or incidence rate, tuberculosis, and HIV-infected persons. Additional searches are also conducted in the free Google search using the reference title (S1 File).

### Eligibility criteria

Just after a manual review of electronic databases, we also used Endnote X8 software to automatically remove exact duplicates. The primary reviewers (TGW and ATM) then conducted a preliminary review based on the title and abstract to remove articles that were clearly unrelated to the study question or did not meet eligibility criteria. The same reviewers then screened the full texts using the pre-specified inclusion and exclusion criteria. Disagreements about eligibility for systematic review and meta-analysis were settled through discussion and a scientifically reasonable common understanding.

### Inclusion and exclusion criteria

The study included studies that assessed tuberculosis incidence rates among HIV-infected people in Sub-Saharan Africa and were published in English between January 1, 2000, and November 25, 2022. Studies that failed to report the overall tuberculosis incidence rate or did not clearly report the number of new tuberculosis cases, editorial commentators, reviews, studies without full text access, publications from a related study, and papers that exclusively reported multi-drug or extensively drug-resistant (MDR or XDR) tuberculosis as outcomes were excluded.

### Extraction of data from eligible papers

Two reviewers worked independently to extract data from selected articles. Discussions were used to resolve disagreements, and if those disagreements continued, another expert colleague was consulted. Using a standardized data extraction form, the following data were extracted for each included study: name of the first author, date of publication, target population, study country, study design, sample size, number of new TB cases, total person-year observation, the incidence rate per 100 person-year observation, and predictors (HR) with their 95% confidence intervals. A spreadsheet created in Microsoft Excel 2007 was used to summarize the data (S2 File).

### Quality assessment for studies

The Joanna Briggs Institute’s (JBI) critical appraisal tools were used to assess the studies quality [53]. The tools were considering the following study characteristics: sampling representativeness and size; identifying confounding and strategies for dealing with it; being free of TB at cohort entry; exposure and outcomes ascertainment during follow-up; a long enough duration of follow-up and participants’ completeness of follow-up; and the appropriate statistical analysis used. Questions that met the required criteria received a score of 1, questions that did not meet the required criteria received a score of 0, and questions not clear enough to meet the required criteria received an unclear (U). The result was graded as low, moderate or high if the quality score was below 49%, 50–69%, or ≥ 70%, respectively. Two independent investigators (TGW and ATM) evaluated the study’s quality, and the inconvenience was resolved by a third colleague expert.

### Operational variables

The dependent variable of this study was the incidence of TB among HIV-positive patients. An incident TB case, or event of interest in this study, is defined as any form of TB diagnosed clinically or radiographically and confirmed by laboratory examinations or by patients who empirically started anti-TB treatment after enrolment [54, 55].

### Data synthesis and statistical analysis

For statistical analysis, the data were exported to STATA version 15. A separate forest plot was calculated to provide the pooled TB incidence rate among HIV-positive persons, along with the 95% confidence interval. Considering the substantial heterogeneity among studies, the random-effects model was used. Different categories were made in order to conduct the subgroup analysis. The Cochran and I^2^ statistics were used to assess heterogeneity [56]. I^2^ statistics were used to assess the magnitude of statistical heterogeneity between studies, and values of 25, 50, and 75% were considered low, medium, and high, respectively. To determine the presence of publication bias, the asymmetry of the funnel plot [57] and the statistical significance of Egger’s regression test (*P*-value < 0.05) were used [58]. Trim-and-fill analysis was used to adjust for publication bias [59]. In addition, we performed a leave-one-out sensitivity analysis to identify the key studies that have a significant effect on between-study heterogeneity. The pooled hazard ratio (HR) is computed based on each factor’s hazard ratio (HR) with 95% confidence intervals (CI) to determine the association between dependent and independent factors. The results were then summarized in the tables and forest plots.

## Results

### Search results

**Fig. 1:**
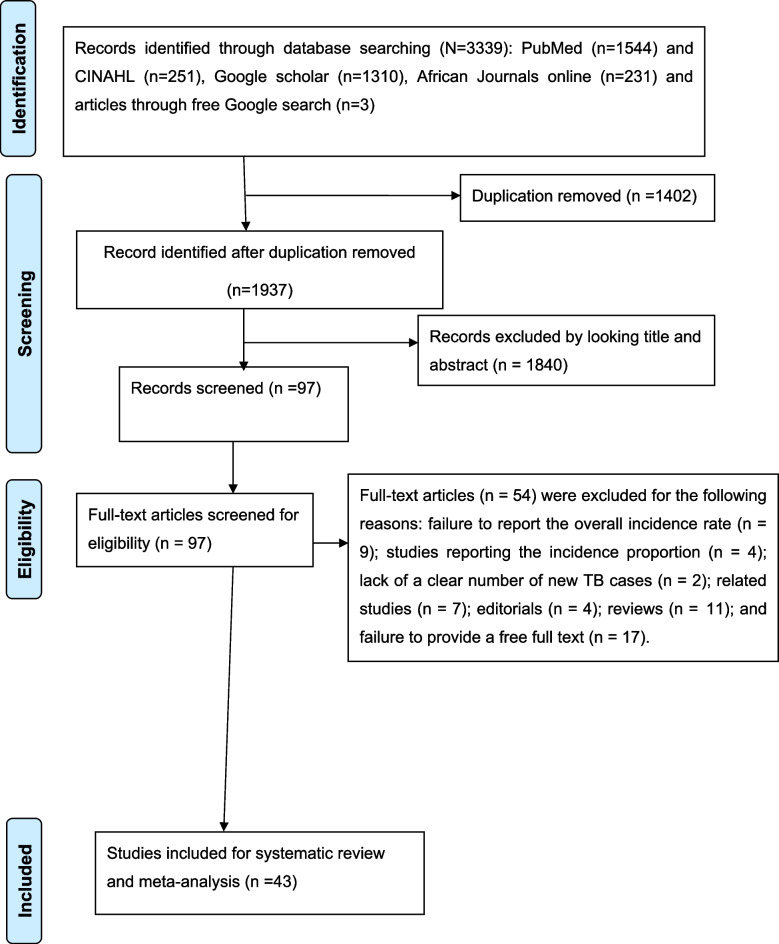
Shows PRISMA flow diagram which shows the selection of articles for systematic review and meta-analysis

The combined database literature search strategies identify 3339 potential studies, with 2 records coming from manual search sources. There were 97 articles left after removing 1402 duplicate articles and 1840 articles because of unrelated titles and abstracts. A total of 97 articles were screened for full-text review, with 54 being rejected for various reasons. Finally, 43 studies were eligible for inclusion in the meta-analysis and systematic review (Fig. 1).

### Description of included studies

Data were collected from 43 studies published between 2000 and 2022 in 13 sub-Saharan African countries. These studies were conducted in four Sub-Saharan African Sub-divisions: East and Central Africa have 27 and 11 studies, respectively, while South and West Africa have eight and seven studies, respectively. Ethiopia, Uganda, and Tanzania have been the most frequently studied countries in East Africa, with Ethiopia having 17 studies, and Uganda and Tanzania each having four. Seven of the eight studies in Africa’s southern region were conducted in South Africa, while three of the studies in western Africa were conducted in Nigeria. Most studies were retrospective cohorts. The majority of the studies included in this review were retrospective and prospective cohort studies, with follow-up periods ranging from one to twelve years. The sample sizes in the included studies range from 212 [60] to 527,249 [61]. The majority of the studies were conducted on adults, with nine of the forty-three studies conducted on children and the remaining four studies conducted on both children and adults. Data were collected from 854,083 HIV-infected persons, and TB was detected in 38,752 of them. The TB incidence rate ranges from 21 to 10,000 person-year in Tanzania [62] to 1050 per 10,000 person-year in South Africa [63] in adults (Table 1).

**Table 1:** Characteristics of included studies and their outcomes (*n* = 43)

ID	African region	Author, publication year	Country	Study design	Follow-up time in year	Study subjects	Sample size	PY	TB cases	IR per 100 PY
1.	East Africa	Ayana GM, et al. [64], 2021	Ethiopia	Retr.	4	Adults	471	18181.82	56	0.31
2.	East Africa	Kebede F, et al. [65], 2021	Ethiopia	Retr.	8	Children	421	1043.1	64	6.1
3.	East Africa	Endalamaw A, et al. [66], 2018	Ethiopia	Retr.	12	Children	352	1294.7	34	2.63
4.	East Africa	Said K, et al. [67], 2017	Tanzania	Retr.	3	Adults	777	2197	18	0.8
5.	East Africa	Alemu A, et al [68], 2020	Ethiopia	Retr	5	Adults	566	2140.08	146	6.82
6.	West Africa	Musa BM, et al. [69], 2015	Nigeria	Retr.	10	Adults	345	2695	47	0.743
7.	East Africa	Aemro A, et al. [70], 2020	Ethiopia	Retr.	5	Adults	494	1000.22	62	6.19
8.	East Africa	Ahamed A, et al. [71], 2017	Ethiopia	Retr.	5	Adults	451	1377.41	119	8.6
9.	South Africa	Hesseling AC, et a l [72], 2009	South Africa	Pros.	3	Children	3321	3320.8	53	1.596
10.	South Africa	Kufa T, et al. [73], 2016	South Africa	Pros.	1	Adults	634	565	15	2.7
11.	West Africa	Jean-François E, et al. [74], 2009	Senegal	Pros.	9.5	Adults	352	1821	42	2.3
12.	East Africa	Getu A, et al. [75], 2022	Ethiopia	Retr.	4.5	Adults	529	1529	74	4.84
13.	West Africa	Wateba MI, et al. [60], 2017	Togo	Pros.	3	Adults	212	636.36	14	2.2
14.	East Africa	Temesgen B, et al. [76], 2019	Ethiopia	Retr.	6	Adults	492	1285.54	83	6.5
15.	West Africa	Pathmanathan I, et al. [77], 2017	Nigeria	Retr.	8	Adults	3072	10,000	57	0.57
16.	East Africa	Tiruneh F, et al. [78], 2020	Ethiopia	Retr.	6	Children	800	2942.99	189	7.917
17.	East Africa	Hermans S.M, et al. [79], 2010	Uganda	Retr.	2	Adults	5982	10,710	336	3.14
18.	East Africa	Majigo M, et al. 61 [61], 2020	Tanzania	Retr.	4	Both	527,249	1,323,600	22,071	1.67
19.	South Africa	Brennan A T, et al. [80], 2016	South Africa	Pros.	2	Both	86,426	121,333	3276	2.7
20.	South Africa	Lawn SD,et al. [81], 2005	South Africa	Pros.	5	Adults	346	1108.8	27	2.44
21.	East Africa	Beshir MT, et al. [82], 2019	Ethiopia	Retr.	5	children	428	1111.1	67	6.03
22.	South Africa	Lawn SD,et al. [63], 2006	South Africa	Pros.	3	Adults	756	782	81	10.5
23.	East Africa	Ayalaw SG, et al. [83], 2015	Ethiopia	Retr.	6	children	271	1100.5	52	4.9
24.	East Africa	Crook AM, et al. [﻿^84^], 2016	Uganda and Zimbabwe	R.C	5	children	969	3632	69	1.9
25.	West Africa	Youngui TB, et al. [85], 2020	West Africa	Retr.	1	Adults	6938	5677.92	189	3.33
26.	East Africa	Mollel EW, et al. [62], 2019	Tanzania	Retr.	6	Adults	78,748	195,296	405	0.21
27.	East Africa	Alemu YM, et al. [86], 2016	Ethiopia	Retr.	5	children	645	1854	79	4.2
28.	East Africa	Dalbo M, et al. [87], 2016	Ethiopia	Retr.	5	Adults	496	1977.6	106	5.36
29.	East Africa	Enju L, et al. [88], 2015	Tanzania	Pros.	8	Adults	67,686	172,773	7602	4.4
30.	South Africa	Bock P, et al. [89], 2019	South Africa	Retr.	2	Adults	2423	2196	97	4.41
31.	East Africa	Alene AK, et al [90], 2013	Ethiopia	Retr.	5	Adults	470	1724.13	136	7.88
32.	South Africa	Gupta A, et al. [91], 2012	South Africa	Pros.	8	Adults	1544	6506	484	7.44
33.	East Africa	Worodria W, et al. [92], 2010	Uganda	Pros.	1	Adults	219	203	14	6.9
34.	East Africa	Bekele H, et al. [93], 2017	Ethiopia	Retr.	6	Adults	554	1830.3	161	8.79
35.	East Africa	Moore D, et al. [94], 2007	Uganda	R.C.	2	Adults	1044	1359	53	3.9
36.	South Africa	Mupfumi L, et al. [95], 2018	Botswana	Retr.	2	Adults	300	428	13	3.04
37.	East Africa	Kebede F, et al. [96], 2021	Ethiopia	Retr.	5	Children	421	10412.15	52	0.499
38.	East Africa	Kazibwe A, et al. [97], 2022	Uganda	Retr.	5	Both	2634	9696.73	22	0.227
39.	East Africa	Fanta A [98], 2020	Ethiopia	Retr.	5	Adults	483	1490	55	3.7
40.	Central Africa	Longo JD, et al [99], 2022	CAR	Retr.	2	Adults	677	1350.7	104	7.7
41.	South Africa	Dembele M, et al. [100], 2010	Burkina Faso	Retr.	8	Adults	2383	7736.8	70	0.905
42.	East Africa	García JI, et al. [101], 2020	Mozambique	Retr.	2	Both	382	685.2	37	5.4
43.	West Africa	Chang CA, et al. [102], 2015	Nigeria	Retr.	6	Adults	50,320	78,228	2021	2.58

*Retr *Retrospective cohort, *Pros *Prospective cohort; and *R.C *Randomized control. Both of which refer to children and adults. *PY* stands for "person-year." *IR* stands for “incidence rate”, *CAR *Central African Republic

### Risk bias assessment for the included studies

Studies with a quality score of 8 or higher were considered to have a low bias. The quality scores of the included studies ranged from 8 to 11. Finally, all 43 included studies have a low-quality bias (≥ 8/11 = 72.7%) (S3 File).

### The pooled incidence rate of TB among HIV-infected persons

**Fig. 2:**
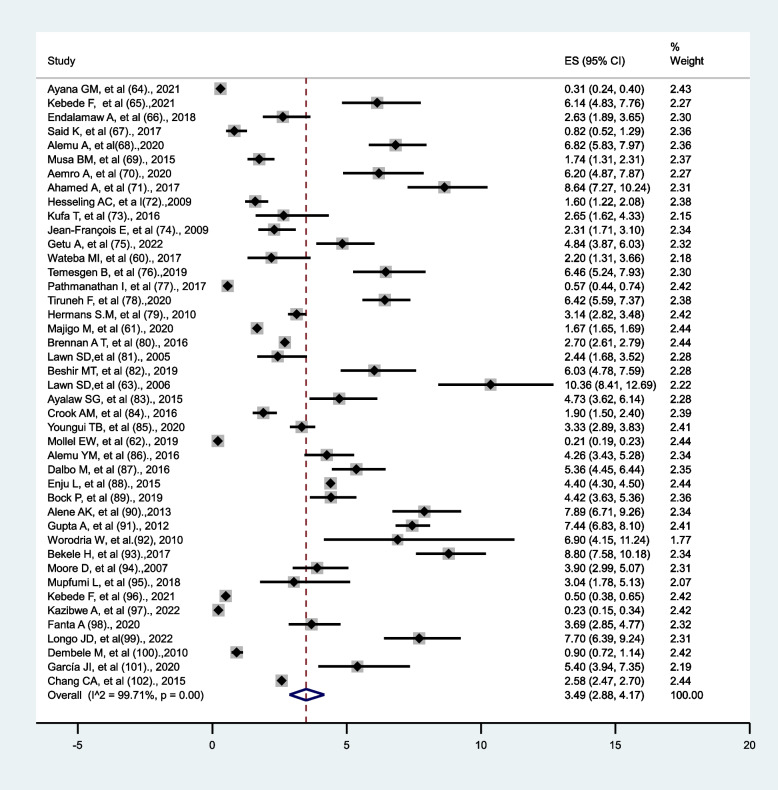
Forest plots of the incidence rate of tuberculosis per 100-person-year observation among HIV-positive persons in Sub-Saharan Africa

Based on the random-effects model, the pooled incidence rate of tuberculosis per 100 person-year observations among HIV-infected persons was 3.49 (95% CI 2.88–4.17), with significant heterogeneity (I^2^ = 99.71%, *p* < 0.001) (Fig. 2).

### Subgroup analyses of tuberculosis incidence rates in HIV-infected persons

Subgroup analysis was conducted based on the predefined categories of African subdivisions, study design, study country, target population, publication year, and length of follow-up time. According to the African division, Central Africa had the highest incidence rate of tuberculosis per 100 person-year among people living with HIV/AIDS, at 7.70 (95% CI: 6.39, 9.24) with no heterogeneity, followed by South Africa at 3.96 (95% CI: 2.43, 5.85) with high significance heterogeneity (I^2^ = 98.35%, *p* < 0.001). However, only one study was conducted in Central Africa. The lowest incidence rate in West Africa was 1.82 (95% CI: 1.02, 2.83), with significant heterogeneity (I^2^ = 98.42%, *p* < 0.001). Similarly, in a country-level subgroup analysis, a single study in the Central African Republic and Burkina Faso revealed the highest and lowest tuberculosis incidence rates, 7.7 per 100 person-year (95% CI: 6.39, 9.24) and 0.9 per 100 person-year (95% CI: 0.72, 1.14), respectively, without heterogeneity. The highest significant heterogeneity was found in Tanzania (I^2^ = 99.97%, *p* < 0.001), Uganda (I^2^ = 99.24%, *p* < 0.001), Ethiopia (I^2^ = 99.16%, *p* < 0.001), and South Africa (I^2^ = 98.59%, *p* < 0.001).

Based on the study design, prospective cohort studies revealed a high tuberculosis incidence rate of 3.81 (95% CI: 2.9, 4.84) per 100 person-year with significant heterogeneity (I^2^ = 99.04%, *p* < 0.001), while retrospective studies revealed a tuberculosis incidence rate of 3.43 (95% CI: 2.73, 4.2) per 100 person-year with significant heterogeneity (I^2^ = 99.63%, *p* < 0.001). The incidence rate of tuberculosis was 3.62 (95% CI: 2.41, 5.08) per 100 person-years for follow-up periods of five years or longer with significant heterogeneity (I^2^ = 99.81%, *p* < 0.001) and 3.23 (95% CI: 2.62, 3.91) per 100 person-years for follow-up periods of less than five years with significant heterogeneity (I^2^ = 98.99%, *p* < 0.001). The pooled TB incidence rate was 3.32 (95% CI: 2.02, 4.93) per 100 person-years, 4.49 (95% CI: 3.24, 5.94) per 100 person-years, 3.29 (95% CI: 1.96, 4.95) per 100 person-years, and 3.42 (95% CI: 2.32, 4.72) per 100 person-years in studies published from 2000 to 2010, 2011 to 2015, 2016–2019, and 2020 to 2022, respectively. The level of heterogeneity in all publication date categories was significantly high (*P* < 0.001). In the subgroup analysis of the target population, four studies included in this systematic review and meta-analysis [59, 78, 95, 99] were excluded from the subgroup analysis because their study populations did not have a distinct age group that differentiated children from adults. In children, the incidence rate of tuberculosis was 3.42 (95% CI: 1.78, 5.57) per 100 person-years, with significant heterogeneity (I^2^ = 98.49%, *p* < 0.001), whereas in adults, the incidence rate was 3.79 (95% CI: 2.63, 5.15) per 100 person-years, with high heterogeneity (I^2^ = 99.78%, *p* < 0.001) (Table 2).

**Table 2:** Tuberculosis incidence rate in HIV-positive people in Sub-Saharan Africa: subgroup meta-analysis and heterogeneity analysis

Categories	Subgroup	IR per 100 PY (95% CI)	Heterogeneity, *p*-value	df
African subdivision	East Africa	3.76(2.87, 4.77)	99.79%, *p* < 0.001	26
	West Africa	1.82 (1.02, 2.83)	98.42%, *p* < 0.001	6
	South Africa	3.96 (2.43, 5.85)	98.35%, *p* < 0.001	7
	Central Africa	7.70 (6.39, 9.24)	-	0
country	Ethiopia	4.84 (2.92,7.22	99.16%, *p* < 0.001	16
	Tanzania	1.42 (0.32, 3.29)	99.97%, *p* < 0.001	3
	Nigeria	1.51(0.38, 3.38)	-	2
	South Africa	4.09 (2.42, 6.17)	98.59%, *p* < 0.001	6
	Senegal	2.31(1.71, 3.1)	-	0
	West Africa	3.33 (2.89, 3.83)	-	0
	Togo	2.20 (1.31, 3.66)	-	0
	Uganda	2.81(0.59, 6.54)	99.24%, *p* < 0.001	3
	Central Africa Republic	7.70 (6.39, 9.24)	-	0
	Uganda and Zimbabwe	1.9 (1.5, 2.4)	-	0
	Burkina Faso	0.9 (0.72, 1.14)	-	0
	Botswana	3.04 (1.78, 5.13)	-	0
	Mozambique	5.4 (3.94, 7.35)	-	0
Study design	Retrospective	3.43 (2.73, 4.2)	99.63%, *p* < 0.001	30
	Prospective	3.81 (2.9, 4.84)	99.04%, *p* < 0.001	9
	Randomized	2.37 (1.96, 2.81)	-	1
Publication year	2000–2010	3.32 (2.02, 4.93)	97.28%, *p* < 0.001	7
	2011–2015	4.49 (3.24, 5.94)	99.38%, *p* < 0.001	5
	2016–2019	3.29 (1.96, 4.95)	99.73%, *p* < 0.001	15
	2020–2022	3.42 (2.32, 4.72)	99.23%, *p* < 0.001	12
Duration of follow-up	≥ 5 years	3.62 (2.41, 5.08)	99.81%, *p* < 0.001	25
	< 5 years	3.23 (2.62, 3.91)	98.99%, *p* < 0.001	16
Target population	Children	3.42 (1.78, 5.57)	98.49%, *p* < 0.001	8
	Adults	3.79 (2.63, 5.15)	99.78%, *p* < 0.001	29

*IR *Incidence rate, *PY *Person-year, *df *degree of freedom

### Publication bias

**Fig. 3:**
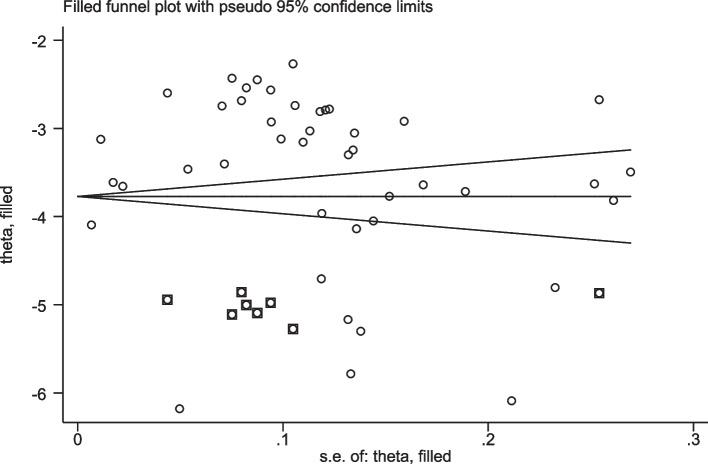
A funnel plot of the pooled incidence rate of tuberculosis in HIV/AIDS patients after meta-trim and fill analysis

The funnel plot and Egger’s regression tests (β_1_ = 0.017 (95% CI: 0.013–0.021), *p* < 0.001) revealed significant publication bias. As a result, the trim-and-fill analysis was performed. The meta-trim-and-fill estimated the number of missing studies to be 8, imputed the omitted studies, and then combined the observed and imputed studies to yield 51 studies. The results of the random meta-trim-and-fill analysis showed that the pooled incidence rate of tuberculosis was − 3.537 (95% CI: -3.745, -3.329) per 100 persons-year for observed studies, and it was − 3.771 (95% CI: -3.970, -3.573) per 100 persons-year for both observed and imputed studies. The imputed studies reduced the pooled incidence rate of tuberculosis. The imputed studies make the funnel plot more symmetrical and highlight areas where studies are missing (Fig. 3).

### Sensitivity analysis

**Fig. 4:**
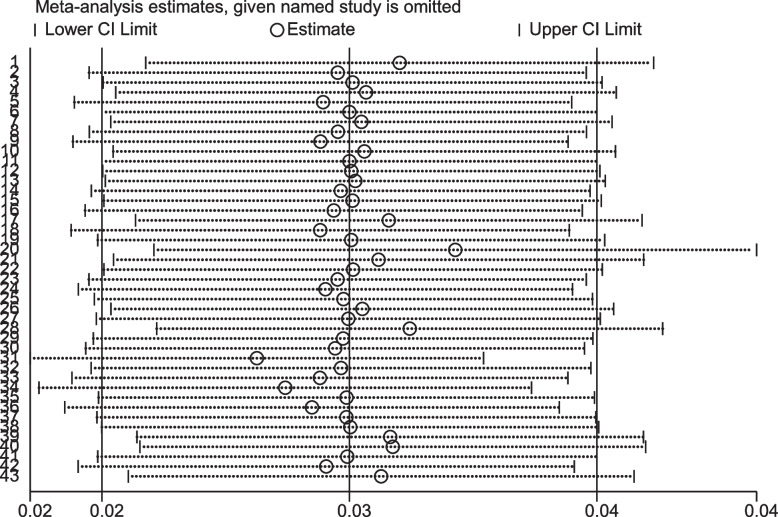
Sensitivity analysis of the tuberculosis incidence rate among HIV-infected persons in sub-Saharan Africa

To investigate potential sources of single-study heterogeneity in the analysis of the incidence rate of tuberculosis in HIV-infected people, a leave-one-out sensitivity analysis was performed. The sensitivity analysis revealed that the findings were not affected by a single study, and all of the leave-one-out point estimates are within the confidence interval of the combined estimate. After removing a single study, our pooled estimated incidence rate ranged between 2.89 (2.26–3.52) and 3.44 (2.6–4.3) per 100 person-year (see Fig. 4).

### Risk factors for tuberculosis among HIV-positive individuals

Significant risk factors for tuberculosis incidence in HIV patients were being underweight, having anemia, being male, having low CD4 counts, being in advanced WHO clinical stages, being bedridden/ambulatory, lacking cotrimoxazole, and lacking IPT. However, antiretroviral therapy (ART) was found to be a protective factor against the occurrence of tuberculosis in HIV patients. A prior history of tuberculosis and level of adherence did not have a significant association with tuberculosis incidence.

### The impact of weight on the occurrence of tuberculosis

**Fig. 5:**
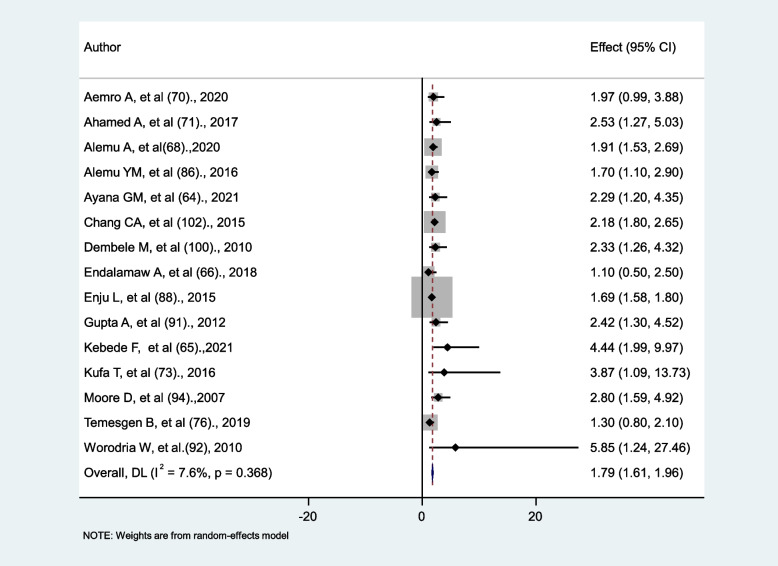
The association between weight and tuberculosis

The findings of fifteen studies were used to determine the relationship between weight and tuberculosis in HIV-infected persons. Two main factors were identified in this analysis, namely underweight and normal. Four studies simply reported using the term “underweight” [65, 66, 76, 86], while the remaining eleven defined it as having a BMI less than 18.5 kg/m^2^. Three studies [66, 70, 76] showed no statistically significant association between being underweight and tuberculosis occurrence, while the rest of the twelve [64, 65, 68, 71, 73, 86, 88, 91, 92, 94, 100, 102] found a significant association. The risk of having tuberculosis for underweight HIV patients was 1.79 times higher compared to normal-weight HIV patients (AHR = 1.79, 95% CI: 1.61, 1.96). There was no significant heterogeneity between studies (I^2^ = 7.6%, *p* = 0.368) (Fig. 5).

### The association between CD4 counts and tuberculosis

**Fig. 6:**
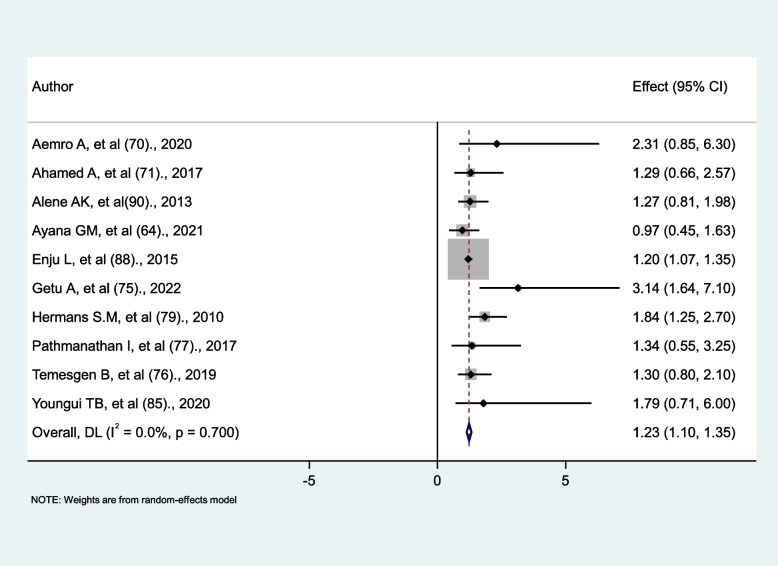
The association between CD4 count and tuberculosis

For this analysis, the two main categories of factors were CD4 counts less than 200 cells/mm^3^ and greater than 200 cells/mm^3^. This cutoff point was selected because the majority of studies analyzed their findings by dividing the CD4 count below and above 200 cells/mm^3^. Furthermore, opportunistic infections occurred when CD4 levels were less than 200 cells/mm^3^. We found ten studies that reported their findings based on this criterion. Only three studies [75, 79, 88] out of ten reported that a low CD4 level (< 200 cells/mm^3^) significantly increased the risk of tuberculosis in HIV-infected persons. Despite the fact that the majority of studies found no significant association between low CD4 levels (< 200 cells/mm^3^) and tuberculosis occurrence, the pooled effect size revealed that low CD4 levels (< 200 cells/mm^3^) were a risk factor for tuberculosis occurrence (AHR = 1.23, 95% CI: 1.1, 1.35) with no evidence of heterogeneity between studies (*p* = 0.7) (Fig. 6).

### Gender differences in tuberculosis occurrence in HIV-infected people

**Fig. 7:**
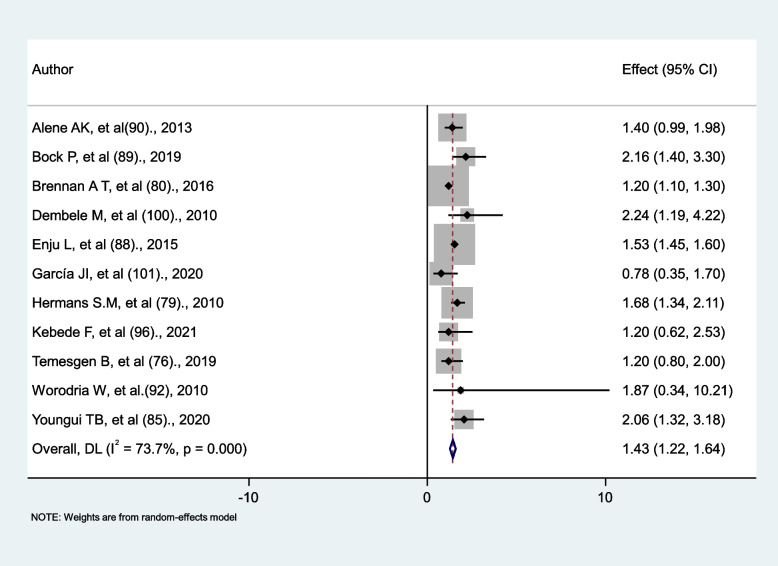
The association between gender and tuberculosis

Eleven primary studies were identified to compare the occurrence of tuberculosis in male HIV patients versus female HIV patients. The association was found to be non-significant in five studies (76, 85, 90, 92, 101,), but significant in six other studies [79, 80, 85, 88, 89, 100]. In this systematic review and meta-analysis, being male was found to be a greater risk factor for developing tuberculosis in HIV patients than being female (AHR = 1.43; 95% CI: 1.22; 1.64) with evidence of heterogeneity (I^2^ = 73.7%, *p* < 0.001) (Fig. 7).

### Determining the association between WHO clinical stages and tuberculosis

**Fig. 8:**
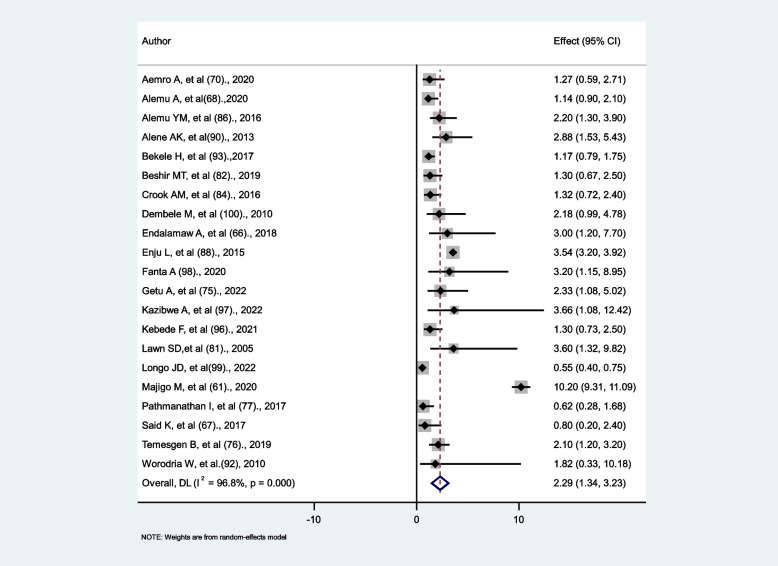
The association between WHO clinical stages and tuberculosis

WHO clinical stages were divided into two categories for statistical analysis: WHO clinical stage ¾ and WHO clinical stage ½ and then stage ¾ was compared with stage½. To assess the relationship between WHO clinical stages and tuberculosis, 21 studies were reviewed. The relationship was found to be positive in 10 studies [61, 66, 75, 76, 81, 86, 88, 90, 97, 98], negative in one study [99], and insignificant in the remaining ten studies. The random effect model meta-analysis revealed that patients with WHO clinical stages ¾ were 2.29 times more likely to develop tuberculosis than patients with WHO clinical stages ½ (AHR = 2.29, 95% CI: 1.34–3.23). There is high heterogeneity between studies (I^2^ = 98.8%, *P* < 0.001) (Fig. 8).

### The association between anemia and tuberculosis

**Fig. 9:**
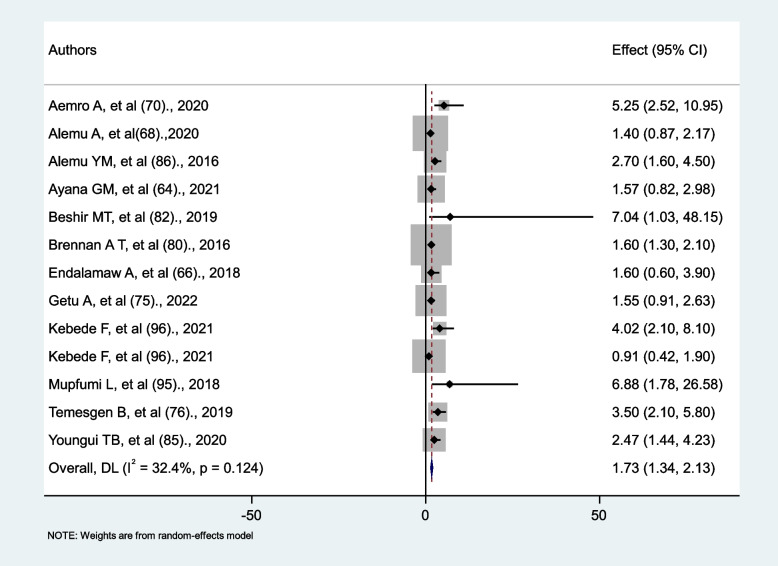
The association between anemia and tuberculosis

Thirteen primary studies were examined in order to determine the relationship between anemia and tuberculosis. All thirteen studies compared hemoglobin (Hgb) levels less than 10 mg/dL with hemoglobin (Hgb) levels ≥ 10 mg/dl. Based on this, two factors were identified for analysis: anemia (Hgb < 10 mg/dl) and non-anemia (Hgb ≥ 10 mg/dl). Five studies found no association between anemia and tuberculosis [64, 66, 68, 75, 96], while the rest of the eight studies found that anemia was a risk factor for tuberculosis. In this meta-analysis, HIV-positive patients with anemia were nearly 2 times more likely to develop tuberculosis than those with non-anemic (AHR = 1.73, 95%CI: 1.34, 2.13). There was no significant heterogeneity across studies (I^2^ = 32.4%, *p* = 0.124) (Fig. 9).

### The effect of functional capacity on tuberculosis occurrence

**Fig. 10:**
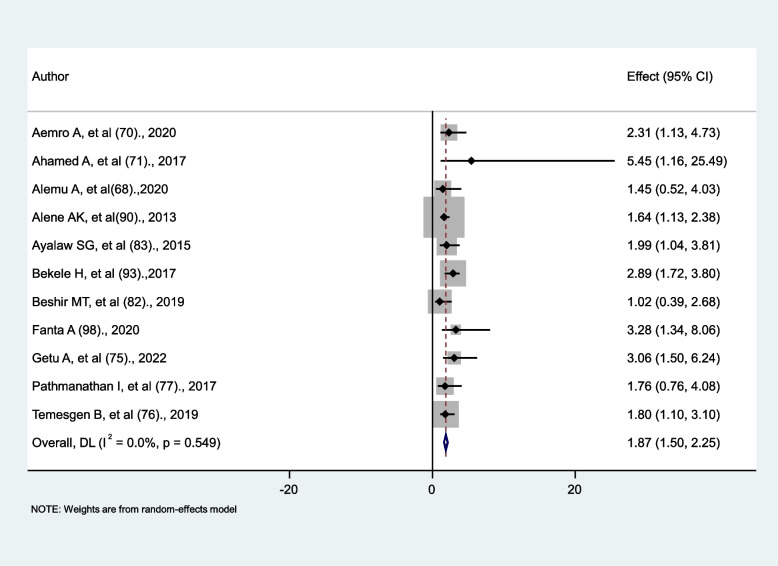
The association between functional capacity and tuberculosis

Eleven studies are used to assess the effect of functional capacity on tuberculosis occurrence. Bedridden/ambulatory and working were the two main factors used in the analysis. The effect was non-significant in three studies [68, 77, 82], but significant in the remaining eight studies [70, 71, 75, 82, 83, 90, 93, 98]. This study revealed that HIV patients who were bedridden/ ambulatory were nearly 1.9 times more likely to develop tuberculosis than HIV patients who were in good health (AHR = 1.87, 95% CI: 1.5, 2.25). Evidence of heterogeneity among studies was absent (I^2^ = 0.0, *p* = 0.549) (Fig. 10).

### The association between prior history tuberculosis and reoccurring tuberculosis

**Fig. 11:**
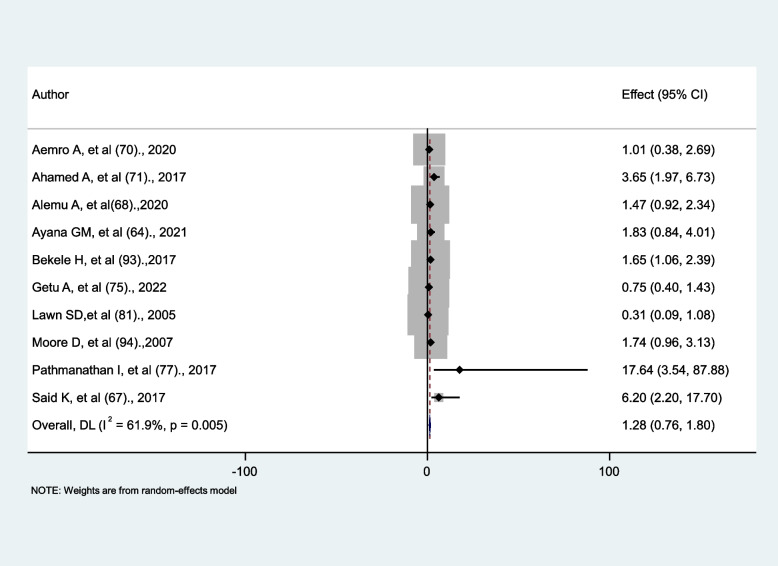
The association between prior history tuberculosis and reoccurring tuberculosis

Ten studies were included to determine the relationship between a prior history of tuberculosis and reoccurring tuberculosis in HIV-infected patients. Four studies [67, 71, 77, 93] show a significant association, while the other six show no significant association. The random effect model in this meta-analysis found no significant association between prior tuberculosis and reoccurring tuberculosis in HIV-positive patients (AHR = 1.28, 95% CI: 0.76, 1.8) with significant heterogeneity (I^2^ = 61.9%, *p* = 0.005) (Fig. 11).

### The effect of antiretroviral treatment (ART) on the occurrence of tuberculosis

**Fig. 12:**
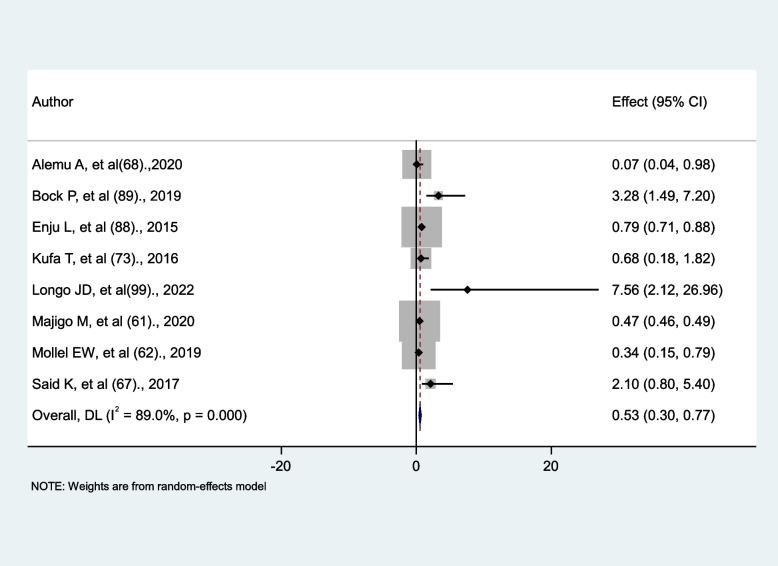
The association between antiretroviral treatment (ART) and tuberculosis

A total of eight studies were evaluated in this meta-analysis to determine whether tuberculosis occurrence increased, decreased, or remained unchanged in antiretroviral therapy (ART) users versus non-users. Two studies [89, 99] reported that tuberculosis increased in antiretroviral therapy (ART) users; four studies reported that tuberculosis decreased in antiretroviral therapy (ART) users [61, 62, 68, 88]; and the remaining two studies reported no significant association [67, 73]. In this random effect meta-analysis model, the risk of tuberculosis is reduced by 47% in antiretroviral therapy (ART) users compared to non-ART users (AHR = 0.53, 95%CI: 0.3, 0.77). There is significant heterogeneity between studies (I^2^ = 89%, *p* < 0.001) (Fig. 12).

### The association between level of adherence and tuberculosis occurrence

**Fig. 13:**
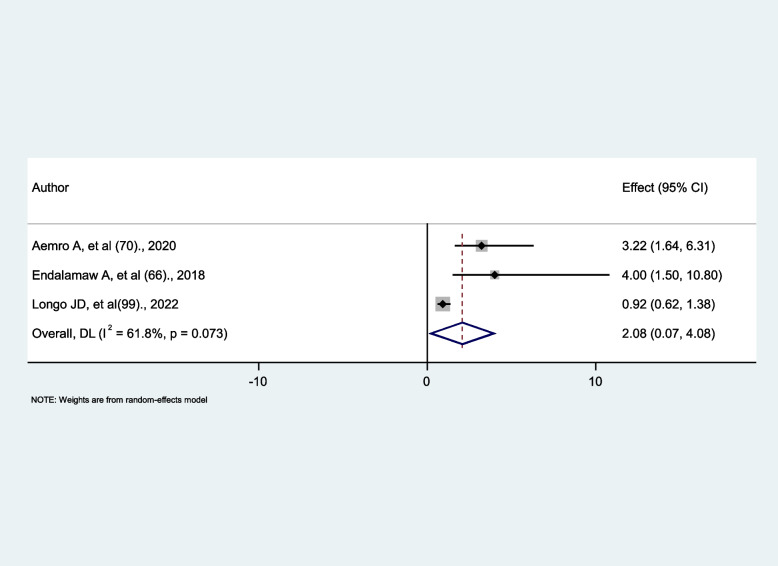
The associations between level of adherence and tuberculosis

We identified three studies [66, 70, 99] that divided adherence into two categories for analysis: good and fair/poor. Two studies’ findings [66, 70] were statistically significant, while just one study’s finding [99] was not. Level of adherence is classified as good when greater or equal to 95% adherence or less than 2 doses missed per month or less than 3 doses missed per 2 months; Otherwise, it can be fair or poor adherence when there is less than or equal to 94% adherence or when there are 2 or more missed doses per month. The findings revealed no significant association between fair/poor adherence and tuberculosis occurrence (AHR = 2.08, 95% CI: 0.07, 4.08), with insignificant high heterogeneity (I^2^ = 61.8%, *p* = 0.073) (Fig. 13).

### The impact of isoniazid prevention therapy (IPT) on the occurrence of tuberculosis

**Fig. 14:**
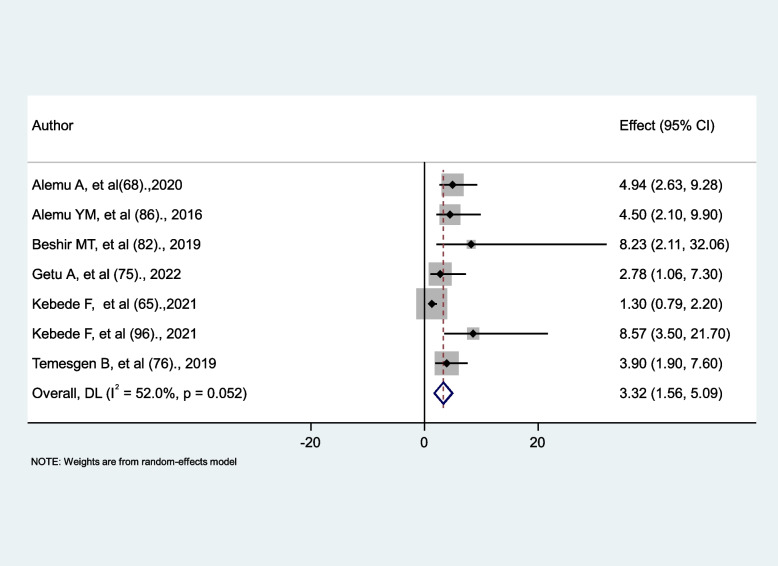
The association between isoniazid prevention therapy (IPT) and tuberculosis

Seven studies [65, 68, 75, 76, 82, 86, 96] were identified to determine the effect of isoniazid prevention therapy (IPT) on tuberculosis occurrence. Six studies revealed a positive association [68, 75, 76, 82, 86, 96], and one study revealed no significant association [65]. The result of meta-analysis showed that HIV patients who did not receive isoniazid prevention therapy (IPT) were nearly three times more likely to develop tuberculosis than those who received IPT (AHR = 3.32, 95% CI: 1.56, 5.09) with insignificant heterogeneity (I^2^ = 52%, *p* = 0.052) (Fig. 14).

### The effect of cotrimoxazole prophylaxis on the occurrence of tuberculosis

**Fig. 15:**
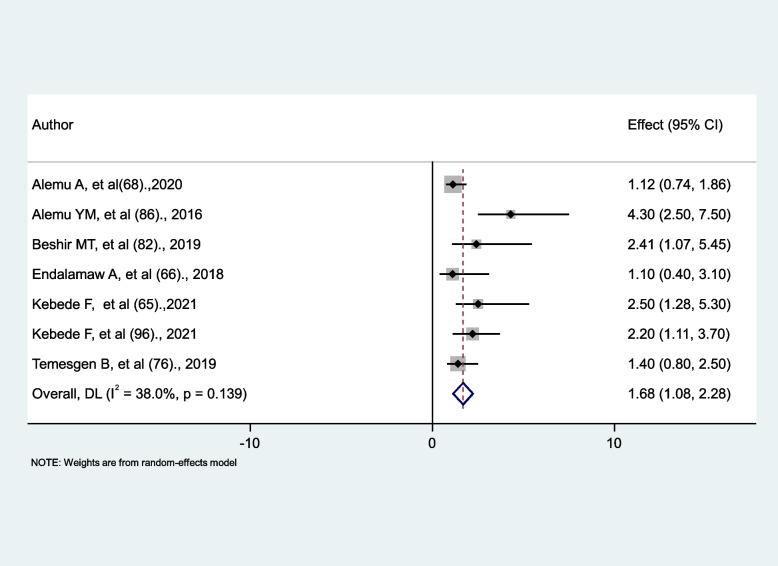
The association between cotrimoxazole prophylaxis and tuberculosis

Seven studies were identified to investigate the association between cotrimoxazole and tuberculosis occurrence. Four studies reported that cotrimoxazole prophylaxis significantly increases the risk of tuberculosis occurrence [65, 82, 86, 96], and three studies reported no significant association [66, 68, 76]. The study’s findings showed that HIV patients without cotrimoxazole prophylaxis had a 1.68 times higher risk of developing tuberculosis as compared to those who took cotrimoxazole prophylaxis (AHR = 1.68, 95% CI: 1.08, 2.28). Moderate heterogeneity with the absence of statistically significant differences between studies was noted (I^2^ = 38%, *p* = 0.139) (Fig. 15).

## Discussion

Studies on tuberculosis incidence among HIV-infected people in various Sub-Saharan African countries were included to determine the pooled incidence rate of tuberculosis in HIV-infected people. This systematic review and meta-analysis study included 43 studies published between 2000 and 2022 in scientific and reputable journals, as well as unpublished articles. The findings of this systematic review and meta-analysis study revealed disparities in the incidence rate of tuberculosis by African subdivision and country level. The study’s lowest rate of tuberculosis was 21 per 10,000 persons per year in Tanzania [62], and the highest rate was 1050 per 10,000 persons per year in South Africa [63]. The findings of this study showed that the pooled incidence rate of tuberculosis among HIV-positive patients was 3.49 per 100 person-years (95% CI: 2.88, 4.17). This result was higher than those from earlier studies conducted in the United States [16], North America and Europe [17], Western and Eastern Europe [18], Spain [19], the United Kingdom [20], Thailand [23] and in low-burden settings [21, 24]. These differences could be attributed to socioeconomic disparities between developed and developing countries, the high burden of HIV/tuberculosis in the study area [25] and the progressive development of latent tuberculosis into active tuberculosis disease, resulting in a tuberculosis epidemic due to the high incidence of HIV [6–9, 13] in the study area in comparison to higher-income countries. They could also be attributed to variations in health system policy and implementation. The findings of the current study are also greater than those of a study among children and adolescents in Sub-Saharan Africa [26], and a prior World Health Organization global tuberculosis report [25]. The discrepancy may be caused by differences in study subjects (this study covered both children and adults) and differences in the concerns of governmental and non-governmental Organizations in TB/HIV prevention and control across countries. The study’s findings were nearly similar to those from Brazil [22], high- or intermediate-burden tuberculosis settings [24], and South Africa [28]. This could be because HIV patients in Brazil may experience virological failure of ART, and similarities with other studies in South Africa and high- or intermediate-burden tuberculosis settings may be due to the same socioeconomic status. However, the current finding was lower than that of a previous study in Sub-Saharan Africa [27], and this difference could be explained by the fact that the previous study in Sub-Saharan Africa used a prospective cohort design and patients did not receive isoniazid prophylaxis, whereas the majority of the current study used retrospective study designs and study subjects who received isoniazid prophylaxis were also included.

In the subgroup analysis African subdivision, patients with HIV in Central and South Africa had the highest incidence rates of tuberculosis, with 7.7 per 100 person-years and 3.96 per 100 person-years, respectively; however, only one study was conducted in Central Africa, whereas patients with HIV in West Africa had the lowest incidence rates of tuberculosis, with 1.82 per 100 person-years. A single study in the Central Republic of Africa [99] had the highest tuberculosis incidence rate, with 7.7 per 100 person-years, whereas another single study in Tanzania [62] had the lowest, with 0.21 per 100 person-years. This variation could be explained by the presence of a single study with an inadequate sample size to determine the outcome and represent these countries, as well as a lack of knowledge among HIV-infected patients about how to cure them and receive follow-up care. Furthermore, the incidence rate of tuberculosis among HIV-infected people in Sub-Saharan Africa remains highly and heterogeneously distributed at the country level (Ethiopia, South Africa, Uganda, and Tanzania) and the African division. The cause of this high and heterogeneous distribution of the tuberculosis incidence rate across these countries and African subdivisions could be inconsistency in addressing the problems due to poorly tracked and incomprehensible strategies, lack of collaboration between donor countries and multilateral institutions to address TB-HIV infection, famine, conflict, and drought. This requires the involvement of international donor organizations, such as WHO and USAIDS, in a collaborative approach that includes dual infection testing and treatment. The incidence rate of tuberculosis among HIV patients was higher with prospective cohort studies [60, 63, 72–74, 80, 81, 88, 91, 92] (3.81 per 100 person-years) than with retrospective studies [61, 62, 64–71, 75–79, 82, 83, 85–87, 89, 90, 93, 95–102] (3.43 per 100 person-years). This could be because prospective studies, which collect data more frequently and require stricter follow-up than retrospective studies, are more reliable at measuring outcomes. A longer period of follow-up (≥ 5 years) had a slightly higher incidence rate of tuberculosis than a shorter period of follow-up (< 5 years), which may be related to a higher occurrence and reporting rate of the outcome. Incidence rates of tuberculosis were 3.32 per 100 persons per year from 2000 to 2010 and 4.49 per 100 persons per year from 2011 to 2015, whereas from 2016 to 2019 and from 2020 to 2022, the incidence rates of tuberculosis were 3.29 and 3.42 per 100 persons per year, respectively. This indicated that tuberculosis is still on the rise and causing serious public health concerns [3–5] and an epidemic in Sub-Saharan Africa, which is consistent with previous reports [7, 9, 14]. The incidence rate of tuberculosis in adults (3.79 per 100 person-years) was slightly higher than in children (3.42 per 100 person-years). This data supports the finding of a previous study, which indicated that the incidence of tuberculosis rises with adult age [15]. This difference in the incidence of tuberculosis between adults and children may be brought on by the challenges of tuberculosis confirmation and diagnosis in children, the lack of systematic data collection on tuberculosis in children, and the fact that young children with tuberculosis are less likely to be infected [15]. The pooled incidence rate of tuberculosis in children was higher than in a previous study of Sub-Saharan [26]. This disparity may be due to HIV patients’ use of health services, such as the timely taking of co-trimoxazole, isoniazid, and ART, and personal feeding behaviors such as eating well-nourished food. The incidence rate of tuberculosis in children revealed the most recent transmission of the disease, particularly a new infection. So its magnitude provides a good proxy measure for tuberculosis transmission in the region. As a result, the high rates of childhood tuberculosis incidence suggest that the region bears a heavy burden of transmission and untreated disease. These findings suggest a need for improved tuberculosis prevention and management in this age group, as well as consideration of their role in tuberculosis epidemics in sub-Saharan Africa. Contact tracing and tuberculosis prevention should be routinely done for children and other HIV patients. The pooled incidence rate of tuberculosis in children was higher than in an earlier study conducted in sub-Saharan Africa [26]. This difference may be due to late diagnosis among HIV patients, and proper use of prophylaxis and ART, which may vary by country and individual. Another distinction could be that HIV and tuberculosis prevention programmes differ by country. In this meta-analysis study, CD4 counts of less than 200 cells/µl, advanced WHO clinical stages (3/4), and being bedridden/ambulatory were risk factors for tuberculosis in HIV patients. This evidence supports previous findings that opportunistic infections, including tuberculosis, are more common in patients with low CD4 levels (under 200 cells/µl) [29–31], bedridden/ambulatory patients, and those in advanced WHO clinical stages [37, 38]. The reason for this could be that the lower the number of CD4 cells, the more severe the disease, as defined by advanced WHO clinical stages and bedridden/ambulatory patients [33]. HIV patients who are also underweight and anemic are more likely to develop tuberculosis than normal. This finding is in line with earlier findings, which found that being underweight [37, 41] and having anemia were strongly associated with HIV disease progression as measured by AIDS [34, 36]. Malnutrition, which weakens immunity and increases tuberculosis reactivation [32], might be the cause of tuberculosis in HIV patients who are underweight and anemic. As a result, HIV patients should receive adequate nutrition as well as long-term financial support. Being male was a significant risk factor for the occurrence of tuberculosis in HIV patients. The rationale may be due to the possibility of social behavior variations and hormonal inequalities between men and women as plausible causes for the TB predominance among adult male patients [103]. This meta-analysis indicated that a lack of taking isoniazid and co-trimoxazole increased the risk of tuberculosis incidence; however, ART users were less likely to develop tuberculosis than ART-none users. These findings back up previous studies that found isoniazid [44, 45] increased the risk of tuberculosis occurrence, but co-trimoxazole use decreased TB occurrence [37, 49], and the absence of ART increased tuberculosis occurrence [39, 40]. In this high-risk region, the use of isoniazid preventive therapy (IPT), co-trimoxazole prophylaxis, and antiretroviral therapy should be scaled up continuously to reduce opportunistic infections like tuberculosis and increase CD4 counts. This study found that poor levels of adherence were not significantly associated with tuberculosis occurrence, which contradicts a previous study [38]. Since a very imprecise confidence interval was noted in this meta-analysis, the small sample size may have contributed to the inability to detect this significant difference.

The majority of the studies are retrospective cohort studies that rely on recorded data that may be incomplete and encompass limited variables. Some studies only had a limited time to follow up with study participants, so the outcome may not have appeared. Subgroup analyses at the African subdivision, in some countries, in study designs, and in the study population revealed significant heterogeneity in tuberculosis incidence rates that could not be fully explained by study variables. Despite these limitations, this study was conducted in high-transmission settings. As a result, it can be used to assess current tuberculosis transmission and burden in HIV patients, as well as plan, implement and evaluate HIV-associated tuberculosis prevention strategies in Sub-Saharan Africa.

## Conclusion

In this systematic review and meta-analysis, the incidence rate of tuberculosis is greater than the WHO African estimation report from 2022. This requires the involvement of donor countries and multilateral institutions such as WHO and USAID in a collaborative effort that includes dual infection testing and treatment. Tuberculosis incidence rate among HIV-positive people varies by country. A well-planned and comprehensible strategy, as well as well-coordinated collaboration across Sub-Saharan countries, should be required to combat tuberculosis incidence and HIV infection. HIV patients should adjust their food consumption in terms of quantity, frequency, and diversity to prevent underweight and anemia. In addition, HIV patients should keep taking antiretroviral therapy, isoniazid, and co-trimoxazole prophylaxis without interruption in order to boost CD4 counts and suppress a high viral load, resulting in recovery from advanced WHO clinical stages and being bedridden/ ambulatory. More research is needed to determine why TB is more common among HIV-positive males than HIV-positive females. In this region, active tuberculosis screening for HIV-positive people should be strengthened routinely.

### Supplementary Information

**Additional file 1.** PRISMA 2020 Checklist.

**Additional file 2: S1 File.** The details of the search strategies for the incidence rate of tuberculosis among HIV-infected persons in Sub-Saharan Africa.

**Additional file 3: S2 File.** data extraction sheet sorted by author, publication year, country, study design, follow-up time, target population, person-year, TB cases and incidence rate.

**Additional file 4: S3 File.** Summary of quality assessments for a cohort study using the JBI appraisal checklist, based on the average rate of two reviewers (TGW and ATM).

## Data Availability

All data generated or analyzed during this study are included in this article.

## Abbreviations

WHO: World health organization
HIV: Human immunodeficiency virus
AIDS: Acquired immunodeficiency syndrome
AOR: Adjusted Odds Ratio
CI: Confidence Interval
S: Supplementary
Fig: Figure
