# Supplementary material for: The incidence rate of tuberculosis and its associated factors among HIV-positive persons in Sub-Saharan Africa: a systematic review and meta-analysis
Source: BMC Infect Dis. 2023 Sep 18;23:613. doi: 10.1186/s12879-023-08533-0 (PMC10507970; doi:10.1186/s12879-023-08533-0)
Supplement: Supplementary file 2 — Additional file 2: S1 File. The details of the search strategies for the incidence rate of tuberculosis among HIV-infected persons in Sub-Saharan Africa. [file 12879_2023_8533_MOESM2_ESM.docx]

S1 File. The details of the search strategies for the incidence rate of tuberculosis among HIV-infected persons in Sub-Saharan Africa

| PubMed | | |
| --- | --- | --- |
| Population | | (Persons[MeSH Terms])) OR (Persons[All Fields])) OR (Person[All Fields])) |
| AND | | |
| Exposure | | (HIV Infections[MeSH Terms])) OR (HIV Infections[All Fields])) OR (HIV Infection[All Fields])) OR (Infection and HIV[All Fields])) OR (Infections and HIV[All Fields])) OR (HTLV-III-LAV Infections[All Fields])) OR (HTLV III LAV Infections[All Fields])) OR (HTLV-III-LAV Infection[All Fields])) OR (Infection and HTLV-III-LAV[All Fields])) OR (Infections and HTLV-III-LAV[All Fields])) OR (T-Lymphotropic Virus Type III Infections and Human[All Fields])) OR (T Lymphotropic Virus Type III Infections and Human[All Fields])) OR (HTLV-III Infections[All Fields])) OR (HTLV III Infections[All Fields])) OR (HTLV-III Infection[All Fields])) OR (Infection and HTLV-III[All Fields])) OR (Infections and HTLV-III[All Fields])) OR (HIV Coinfection[All Fields])) OR (Coinfection and HIV[All Fields])) OR (Coinfections and HIV[All Fields])) OR (HIV Coinfections[All Fields])) |
| AND | | |
| Outcome | (Incidences [MeSH Terms])) OR (Incidences[All Fields])) OR (Incidence Rate [All Fields])) OR (Incidence Rates[All Fields])) OR (Rate and Incidence[All Fields])) OR (Person-time Rate[All Fields])) OR (Person time Rate[All Fields])) OR (Person-time Rates[All Fields])) OR (Rate and Person-time[All Fields]))) | |
|  | AND | |
|  | (Tuberculosis[MeSH Terms])) OR (Tuberculosis[All Fields])) OR (Tuberculoses[All Fields])) OR (Kochs Disease[All Fields])) OR (Koch's Disease[All Fields])) OR (Koch Disease[All Fields])) OR (Mycobacterium tuberculosis Infection[All Fields])) OR (Infection and Mycobacterium tuberculosis) [All Fields]) OR (Infections and Mycobacterium tuberculosis[All Fields])) OR (Mycobacterium tuberculosis Infections[All Fields])) | |
| AND | | |
| Context | (Africa South of the Sahara[MeSH Terms])) OR (Africa South of the Sahara[All Fields])) OR (Subsaharan Africa[All Fields])) OR (Africa and Sub-Saharan[All Fields])) OR (Sub-Saharan Africa[All Fields]) | |
| Filters | Humans, English langusge, Free full text, publication date from 01/01/2022 -25/11/2022 | |
| CINAHL | | |
| Population | (Persons) [Subject Terms] OR (persons) [All Fields] | |
| AND | | |
| Exposure | (HIV Infections) [Subject Terms] | |
| AND | | |
| Outcome | Incidence [Subject Terms] OR (Incidence rate) [All Fields] | |
|  | AND | |
|  | Tuberculosis [Subject Terms] | |
| AND | | |
| Context | Sub-Saharan Africa [All Fields] | |
| Filter | English language, and publication dates ranging from January 1, 2000, to November 25, 2022, with open access full text. | |
| Google scholar | | |
| Population | Persons | |
| AND | | |
| Exposure | “HIV-infected” | |
| AND | | |
| Outcome | Tuberculosis | |
| AND | | |
| Context | “Sub-Saharan Africa” | |
| filter | English language, publication date: 2000 – 2022 | |
| Online African Journals  The following specific search terms were entered into the Google custom search text box: | | |
| Population | Persons | |
| Exposure | HIV infection | |
| Outcome | incidence or incidence rate, tuberculosis | |
| Additional searches are also conducted in the free Google search using the reference title | | |
