# Supplementary material for: The incidence rate of tuberculosis and its associated factors among HIV-positive persons in Sub-Saharan Africa: a systematic review and meta-analysis
Source: BMC Infect Dis. 2023 Sep 18;23:613. doi: 10.1186/s12879-023-08533-0 (PMC10507970; doi:10.1186/s12879-023-08533-0)
Supplement: Supplementary file 3 — Additional file 3: S2 File. data extraction sheet sorted by author, publication year, country, study design, follow-up time, target population, person-year, TB cases and incidence rate. [file 12879_2023_8533_MOESM3_ESM.docx]

| S2 File. data extraction sheet sorted by author, publication year, country, study design, follow-up time, target population, person-year, TB cases and incidence rate | | | | | | | | | |
| --- | --- | --- | --- | --- | --- | --- | --- | --- | --- |
| Study ID | Author, publication year | Country | Study design | Follow-up time in year | Study subjects | Sample size | PY | TB cases | IR per 100 PY |
|  | Ayana GM, et al (64)., 2021 | Ethiopia | Retrospective cohort | 4 | Adults | 471 | 18181.82 | 56 | 0.31 |
|  | Kebede F, et al (65).,2021 | Ethiopia | Retrospective cohort | 8 | Children | 421 | 1043.1 | 64 | 6.1 |
|  | Endalamaw A, et al (66)., 2018 | Ethiopia | Retrospective cohort | 12 | Children | 352 | 1294.7 | 34 | 2.63 |
|  | Said K, et al (67)., 2017 | Tanzania | Retrospective cohort | 3 | Adults | 777 | 2197 | 18 | 0.8 |
|  | Alemu A, et al(68).,2020 | Ethiopia | Retr | 5 | Adults | 566 | 2140.08 | 146 | 6.82 |
|  | Musa BM, et al (69)., 2015 | Nigeria | Retrospective cohort | 10 | Adults | 345 | 2695 | 47 | 0.743 |
|  | Aemro A, et al (70)., 2020 | Ethiopia | Retrospective cohort | 5 | Adults | 494 | 1000.22 | 62 | 6.19 |
|  | Ahamed A, et al (71)., 2017 | Ethiopia | Retrospective cohort | 5 | Adults | 451 | 1377.41 | 119 | 8.6 |
|  | Hesseling AC, et a l(72)., 2009 | South Africa | Pros. | 3 | Children | 3321 | 3320.8 | 53 | 1.596 |
|  | Kufa T, et al (73)., 2016 | South Africa | Prospective cohort | 1 | Adults | 634 | 565 | 15 | 2.7 |
|  | Jean-François E, et al (74)., 2009 | Senegal | Prospective cohort | 9.5 | Adults | 352 | 1821 | 42 | 2.3 |
|  | Getu A, et al (75)., 2022 | Ethiopia | Retrospective cohort | 4.5 | Adults | 529 | 1529 | 74 | 4.84 |
|  | Wateba MI, et al (60)., 2017 | Togo | Prospective cohort | 3 | Adults | 212 | 636.36 | 14 | 2.2 |
|  | Temesgen B, et al (76)., 2019 | Ethiopia | Retrospective cohort | 6 | Adults | 492 | 1285.54 | 83 | 6.5 |
|  | Pathmanathan I, et al (77)., 2017 | Nigeria | Retrospective cohort | 8 | Adults | 3072 | 10000 | 57 | 0.57 |
|  | Tiruneh F, et al (78).,2020 | Ethiopia | Retrospective cohort | 6 | Children | 800 | 2942.99 | 189 | 7.917 |
|  | Hermans S.M, et al (79)., 2010 | Uganda | Retrospective cohort | 2 | Adults | 5982 | 10710 | 336 | 3.14 |
|  | Majigo M, et al (61)., 2020 | Tanzania | Retrospective cohort | 4 | children & adults | 527249 | 1323600 | 22071 | 1.67 |
|  | Brennan A T, et al (80)., 2016 | South Africa | Prospective cohort | 2 | children & adults | 86426 | 121333 | 3276 | 2.7 |
|  | Lawn SD,et al (81)., 2005 | South Africa | Prospective cohort | 5 | Adults | 346 | 1108.8 | 27 | 2.44 |
|  | Beshir MT, et al (82)., 2019 | Ethiopia | Retrospective cohort | 5 | children | 428 | 1111.1 | 67 | 6.03 |
|  | Lawn SD,et al (63)., 2006 | South Africa | Prospective cohort | 3 | Adults | 756 | 782 | 81 | 10.5 |
|  | Ayalaw SG, et al (83)., 2015 | Ethiopia | Retrospective cohort | 6 | children | 271 | 1100.5 | 52 | 4.9 |
|  | Crook AM, et al (84)., 2016 | Uganda & Zimbabwe | Randomized control | 5 | children | 969 | 3632 | 69 | 1.9 |
|  | Youngui TB, et al (85)., 2020 | West Africa | Retrospective cohort | 1 | Adults | 6938 | 5677.92 | 189 | 3.33 |
|  | Mollel EW, et al (62)., 2019 | Tanzania | Retrospective cohort | 6 | Adults | 78748 | 195296 | 405 | 0.21 |
|  | Alemu YM, et al (86)., 2016 | Ethiopia | Retrospective cohort | 5 | children | 645 | 1854 | 79 | 4.2 |
|  | Dalbo M, et al (87)., 2016 | Ethiopia | Retrospective cohort | 5 | Adults | 496 | 1977.6 | 106 | 5.36 |
|  | Enju L, et al (88)., 2015 | Tanzania | Prospective cohort | 8 | Adults | 67686 | 172773 | 7602 | 4.4 |
|  | Bock P, et al (89)., 2019 | South Africa | Retrospective cohort | 2 | Adults | 2423 | 2196 | 97 | 4.41 |
|  | Alene AK, et al(90)., 2013 | Ethiopia | Retrospective cohort | 5 | Adults | 470 | 1724.13 | 136 | 7.88 |
|  | Gupta A, et al (91)., 2012 | South Africa | Prospective cohort | 8 | Adults | 1544 | 6506 | 484 | 7.44 |
|  | Worodria W, et al.(92), 2010 | Uganda | Prospective cohort | 1 | Adults | 219 | 203 | 14 | 6.9 |
|  | Bekele H, et al (93).,2017 | Ethiopia | Retrospective cohort | 6 | Adults | 554 | 1830.3 | 161 | 8.79 |
|  | Moore D, et al (94).,2007 | Uganda | Randomized control | 2 | Adults | 1044 | 1359 | 53 | 3.9 |
|  | Mupfumi L, et al (95)., 2018 | Botswana | Retrospective cohort | 2 | Adults | 300 | 428 | 13 | 3.04 |
|  | Kebede F, et al (96)., 2021 | Ethiopia | Retrospective cohort | 5 | Children | 421 | 10412.15 | 52 | 0.499 |
|  | Kazibwe A, et al (97)., 2022 | Uganda | Retrospective cohort | 5 | children & adults | 2634 | 9696.73 | 22 | 0.227 |
|  | Fanta A (98)., 2020 | Ethiopia | Retrospective cohort | 5 | Adults | 483 | 1490 | 55 | 3.7 |
|  | Longo JD, et al(99)., 2022 | CAR | Retrospective cohort | 2 | Adults | 677 | 1350.7 | 104 | 7.7 |
|  | Dembele M, et al (100)., 2010 | Burkina Faso | Retrospective cohort | 8 | Adults | 2383 | 7736.8 | 70 | 0.905 |
|  | García JI, et al (101)., 2020 | Mozambique | Retrospective cohort | 2 | children & adults | 382 | 685.2 | 37 | 5.4 |
|  | Chang CA, et al (102)., 2015 | Nigeria | Retrospective cohort | 6 | Adults | 50320 | 78228 | 2021 | 2.58 |

PY=Person-Year, IR=Incidence Rate, TB=Tuberculosis
