# Supplementary material for: The incidence rate of tuberculosis and its associated factors among HIV-positive persons in Sub-Saharan Africa: a systematic review and meta-analysis
Source: BMC Infect Dis. 2023 Sep 18;23:613. doi: 10.1186/s12879-023-08533-0 (PMC10507970; doi:10.1186/s12879-023-08533-0)
Supplement: Supplementary file 4 — Additional file 4: S3 File. Summary of quality assessments for a cohort study using the JBI appraisal checklist, based on the average rate of two reviewers (TGW and ATM). [file 12879_2023_8533_MOESM4_ESM.docx]

S3 File. . Summary of quality assessments for a cohort study using the JBI appraisal checklist, based on the average rate of two reviewers (TGW and ATM).

| Author, publication year | Q1 | Q2 | Q3 | Q4 | Q5 | Q6 | Q7 | Q8 | Q9 | Q10 | Q11 | Raw score (%) | Risk |
| --- | --- | --- | --- | --- | --- | --- | --- | --- | --- | --- | --- | --- | --- |
| Ayana GM, et al (64)., 2021 | 1 | 1 | 1 | 1 | 1 | 1 | 1 | 0 | 1 | 1 | 1 | 10/11 (90.1) | low |
| Kebede F, et al (65).,2021 | 1 | 1 | 1 | 1 | 1 | 1 | 1 | 1 | 1 | 1 | 1 | 11/11 (100) | low |
| Endalamaw A, et al (66)., 2018 | 1 | 1 | 1 | 1 | 1 | 1 | 1 | 1 | 1 | 1 | 1 | 11/11 (100) | low |
| Said K, et al (67)., 2017 | 1 | 1 | 1 | 1 | 1 | 1 | 1 | 0 | 1 | 1 | 1 | 10/11 (90.1) | low |
| Alemu A, et al(68).,2020 | 1 | 1 | 1 | 1 | 1 | 1 | 1 | 1 | 1 | 1 | 1 | 11/11 (100) | low |
| Musa BM, et al (69)., 2015 | 1 | 1 | 1 | 1 | 1 | 1 | 1 | 1 | U | 1 | 1 | 9/11 (81.9%), | low |
| Aemro A, et al (70)., 2020 | 1 | 1 | 1 | 1 | 1 | 1 | 1 | 1 | 1 | 1 | 1 | 11/11 (100) | low |
| Ahamed A, et al (71)., 2017 | 1 | 1 | 1 | 1 | 1 | 1 | 1 | 1 | 1 | 1 | 1 | 11/11 (100) | low |
| Hesseling AC, et a l(72)., 2009 | 1 | 1 | 1 | 1 | 1 | 1 | 1 | 0 | 1 | 1 | 1 | 10/11 (90.1) | low |
| Kufa T, et al (73)., 2016 | 1 | 1 | 1 | 1 | 1 | 1 | 1 | 1 | 1 | 1 | 1 | 11/11 (100) | low |
| Jean-François E, et al (74)., 2009 | 1 | 1 | 1 | 0 | 0 | 1 | 1 | 1 | 1 | 1 | 1 | 9/11 (81.9) | low |
| Getu A, et al (75)., 2022 | 1 | 1 | 1 | 1 | 1 | 1 | 1 | 1 | 1 | 1 | 1 | 11/11 (100) | low |
| Wateba MI, et al (60)., 2017 | 1 | 1 | 1 | 0 | 0 | 1 | 1 | 1 | 0 | 1 | 1 | 8/11 (72.3) | low |
| Temesgen B, et al (76)., 2019 | 1 | 1 | 1 | 1 | 1 | 1 | 1 | 1 | 1 | 1 | 1 | 11/11 (100) | low |
| Pathmanathan I, et al (77)., 2017 | 1 | 1 | 1 | 1 | 1 | 1 | 1 | 1 | 1 | 1 | 1 | 11/11 (100) | low |
| Tiruneh F, et al (78).,2020 | 1 | 1 | 1 | 1 | 1 | 1 | 1 | 1 | 1 | 1 | 1 | 11/11 (100) | low |
| Hermans S.M, et al (79)., 2010 | 1 | 1 | 1 | 1 | 1 | 1 | 1 | 1 | 1 | 1 | 1 | 11/11 (100) | low |
| Majigo M, et al (61)., 2020 | 1 | 1 | 1 | 1 | 1 | 1 | 1 | 1 | 1 | 1 | 1 | 11/11 (100) | low |
| Brennan A T, et al (80)., 2016 | 1 | 1 | 1 | 1 | 1 | 1 | 1 | 0 | 0 | 1 | 1 | 9/11 (81.9) | low |
| Lawn SD,et al (81)., 2005 | 1 | 1 | 1 | 1 | 1 | 1 | 1 | 1 | 1 | 1 | 1 | 11/11 (100) | low |
| Beshir MT, et al (82)., 2019 | 1 | 1 | 1 | 1 | 1 | 1 | 1 | 1 | 1 | 1 | 1 | 11/11 (100) | low |
| Lawn SD,et al (63)., 2006 | 1 | 1 | 1 | 1 | 1 | 1 | 1 | 0 | 1 | 1 | 1 | 10/11 (90.9) | low |
| Ayalaw SG, et al (83)., 2015 | 1 | 1 | 1 | 1 | 1 | 1 | 1 | 1 | 1 | 1 | 1 | 11/11 (100) | low |
| Crook AM, et al (84)., 2016 | 1 | 1 | 1 | 1 | 1 | 1 | 1 | 1 | 1 | 1 | 1 | 11/11 (100) | low |
| Youngui TB, et al (85)., 2020 | 0 | 1 | 1 | 1 | 1 | 1 | 1 | 1 | 0 | 1 | 1 | 9/11 (81.9) | low |
| Mollel EW, et al (62)., 2019 | 1 | 1 | 1 | 1 | 1 | 1 | 1 | 1 | 1 | 1 | 1 | 11/11 (100) | low |
| Alemu YM, et al (86)., 2016 | 1 | 1 | 1 | 1 | 1 | 1 | 1 | 1 | 1 | 1 | 1 | 11/11 (100) | low |
| Dalbo M, et al (87)., 2016 | 1 | 1 | 1 | 1 | 1 | 1 | 1 | 1 | 1 | 1 | 1 | 11/11 (100) | low |
| Enju L, et al (88)., 2015 | 1 | 1 | 1 | 1 | 1 | 1 | 1 | 1 | 1 | 1 | 1 | 11/11 (100) | low |
| Bock P, et al (89)., 2019 | 1 | 1 | 1 | 1 | 1 | 1 | 1 | 0 | 0 | 1 | 1 | 10/11 (90.9) | low |
| Alene AK, et al(90)., 2013 | 1 | 1 | 1 | 1 | 1 | 1 | 1 | 1 | 1 | 1 | 1 | 11/11 (100) | low |
| Gupta A, et al (91)., 2012 | 1 | 1 | 1 | 1 | 1 | 1 | 1 | 1 | 0 | 1 | 1 | 10/11 (90.9) | low |
| Worodria W, et al.(92), 2010 | 1 | 1 | 1 | 1 | 1 | 1 | 1 | 0 | 1 | 1 | 1 | 10/11 (90.9) | low |
| Bekele H, et al (93).,2017 | 1 | 1 | 1 | 1 | 1 | 1 | 1 | 1 | U | 1 | 1 | 10/11 (90.9) | low |
| Moore D, et al (94).,2007 | 1 | 1 | 1 | 1 | 1 | 1 | 1 | 0 | U | 1 | 1 | 9/11 (81.9) | low |
| Mupfumi L, et al (95)., 2018 | 1 | 1 | 1 | 1 | 1 | 1 | 1 | 0 | 1 | 1 | 1 | 10/11 (90.9) | low |
| Kebede F, et al (96)., 2021 | 1 | 1 | 1 | 1 | 1 | 1 | 1 | 1 | 1 | 1 | 1 | 11/11 (100) | low |
| Kazibwe A, et al (97)., 2022 | 1 | 1 | 1 | 1 | 1 | 1 | 1 | 1 | 1 | 1 | 1 | 11/11 (100) | low |
| Fanta A (98)., 2020 | 1 | 1 | 1 | 1 | 1 | 1 | 1 | 1 | 1 | 1 | 1 | 11/11 (100) | low |
| Longo JD, et al(99)., 2022 | 1 | 1 | 1 | 1 | 1 | 1 | 1 | 0 | U | 1 | 1 | 9/11 (81.8) | low |
| Dembele M, et al (100)., 2010 | 1 | 1 | 1 | 1 | 1 | 1 | 1 | 1 | 1 | 1 | 1 | 11/11 (100) | low |
| García JI, et al (101)., 2020 | 1 | 1 | 1 | 1 | 1 | 1 | 1 | 0 | U | 1 | 1 | 9/11 (81.8) | low |
| Chang CA, et al (102)., 2015 | 1 | 1 | 1 | 1 | 1 | 1 | 1 | 1 | 0 | 1 | 1 | 10/11 (90.9) | low |

**JBI Critical Appraisal Checklist for cohort studies to be scored:**

Q1. Were the two groups similar and recruited from the same population

Q2. Were the exposures measured similarly to assign people to both exposed and unexposed groups?

Q3. Was the exposure measured in a valid and reliable way?

Q4. Were confounding factors identified?

Q5. Were strategies to deal with confounding factors stated?

Q6. Were the groups/participants free of the outcome at the start of the study (or at the moment of exposure?)

Q7. Were the outcomes measured in a valid and reliable way?

Q8. Was the follow up time reported and sufficient to be long enough for outcomes to occur?

Q9. Was follow up complete, and if not, were the reasons to loss to follow up described and explored?

Q10. Were strategies to address incomplete follow up utilized?

Q11. Was appropriate statistical analysis used?

**Abbreviations:** 1 = yes; 0 = No; U = Unclear; NA = Not Applicable; JBI: Joanna

Briggs Institute

**Criteria used to rank the risk of bias:**

i) ≤49% = high risk of Bias

ii) 50% and 69% = Moderate risk of Bias

iii) Above 70% = low risk of Bias
